# MMP-9 inhibitors impair learning in spontaneously hypertensive rats

**DOI:** 10.1371/journal.pone.0208357

**Published:** 2018-12-11

**Authors:** Limor Raz, Yi Yang, Jeffrey Thompson, Sasha Hobson, John Pesko, Shahriar Mobashery, Mayland Chang, Gary Rosenberg

**Affiliations:** 1 Department of Neurology, University of New Mexico, Albuquerque, New Mexico, United States of America; 2 UNM Memory and Aging Center, University of New Mexico, Albuquerque, New Mexico, United States of America; 3 AbbVie, Data and Statistical Sciences, North Chicago, Illinois, United States of America; 4 Department of Chemistry and Biochemistry, University of Notre Dame, Notre Dame, Indiana, United States of America; Massachusetts General Hospital/Harvard Medical School, UNITED STATES

## Abstract

Vascular cognitive impairment dementia (VCID) is a major cause of cognitive loss in the elderly. Matrix metalloproteinases (MMPs) are a family of proteases involved in remodeling the extracellular matrix in development, injury and repair. Blood-brain barrier (BBB) disruption due to inflammation mediated by MMPs is a mechanism of white matter injury. Currently there are no treatments besides the control of vascular risk factors. We tested two MMP-9 inhibitors that improved outcome in acute stroke: DP-460 and SB-3CT. We hypothesized that these inhibitors would have a beneficial effect in chronic stroke by reducing edema in white matter and improving behavioral outcomes. Spontaneously hypertensive stroke-prone rats (SHRSPs) with unilateral carotid artery occlusion (UCAO) fed a Japanese Permissive Diet (JPD) were used as a model of VCID. JPD was begun in the 12^th^ week of life. Rats were treated with DP-460 (500 mg/kg) for 4 weeks, or SB-3CT (10 mg/kg) for 8 weeks, beginning at the UCAO/JPD onset. Rats treated with a dextrose or DMSO solution served as vehicle controls. Naïve SHRSPs on a standard diet served as sham control. Magnetic resonance imaging (MRI) analyses of the corpus callosum, external capsule, hippocampus and Morris water maze behavioral tests were conducted. We found an increase in body weight (p = 0.004) and blood pressure (p = 0.007) at 15 weeks with the DP-460 drug. SB-3CT increased body weight at 14 weeks (p = 0.015) and had significant but variable effects on blood pressure. Neither drug affected imaging parameters. Behavioral studies showed an impaired ability to learn with DP-460 (p<0.001) and no effect on learning with SB-3CT. Unchanged MMP-9 levels were detected in DP-460-treated rats via gel zymography. Our findings suggest that MMPs are not major factors in white matter damage in the SHRSP model of VCID and that drugs that are relatively selective for MMP-9 can interfere with learning.

## Introduction

Vascular cognitive impairment dementia (VCID) is an important cause of dementia and an accelerator of cognitive decline in Alzheimer’s disease (AD).[1, 2] Neuroinflammation is a major contributor to progressive injury to the white matter.[3, 4] Earlier, we showed that minocycline blocks the damage to the white matter and improves cognition in spontaneously hypertensive stroke-prone rats (SHRSPs), which is an animal model of VCID.[5, 6] Blood-brain barrier (BBB) disruption mediated by pro-inflammatory matrix metalloproteinases (MMPs) is a major factor in the mechanism of white matter injury.[7, 8] Previously, we have shown that SHRSPs subjected to unilateral carotid artery occlusion (UCAO) and Japanese permissive diet (JPD), underwent white matter injury that is associated with BBB disruption.[9] This BBB disruption occurred secondary to increased hypoxia-inducible factor-1α (HIF-1α), a transcriptional regulator elevated during hypoxia, which induced an MMP-9-mediated infiltration of leukocytes.[7] The observed improvements in brain structure and function with minocycline could have been due to an indirect reduction in inflammation by blocking microglia activity or by directly blocking the action of MMP-9.

To resolve this controversy, we studied the impact of two MMP-9 inhibitors in the same animal model that was used to study minocycline. Both inhibitors, DP-460 and SB-3CT, cross the BBB and have been shown to exert beneficial effects in neurological diseases, including acute stroke and amyotrophic lateral sclerosis, but have not been tested in a model of VCID.[10, 11] We used SHRSP fed a high salt, low protein JPD to test the effect of the two drugs on white matter injury with MRI and behavior with the Morris Water Maze (MWM).[12, 13] We hypothesized that MMP-9 inhibition was the reason for the beneficial effects of minocycline. To test the hypothesis, we obtained two MMP inhibitors, DP-460, an active-site zinc ion chelator, and SB-3CT, a slow-binding inhibitor of MMP-9 that undergoes a reaction within the active site to manifest tight-binding behavior.[14]

## Methods

### Animals

All animal studies were reviewed and approved by the Institutional Committee on Animal Use and Care (15-200248-HSC) at the University of New Mexico in accordance with institutional guidelines. Male SHRSP rats were purchased from Charles River Laboratories. Methods for the UCAO surgery, JPD and blood pressure measurements have been reported.[9] Briefly, SHRSPs were purchased at 6 weeks of age. The high salt, low protein diet was administered and UCAO surgery performed at 12 weeks of age. In the UCAO group, the right carotid artery was isolated and double-ligated permanently with 4–0 silk sutures under deep anesthesia with 2.0% isoflurane. Following UCAO, rats were placed on the JPD (16% protein, 0.75% potassium, 4% sodium; Ziegler Bros, Inc.) with 1% sodium chloride added to the drinking water. At the end of the study, animals were deeply anesthetized with sodium pentobarbital and transcardially perfused with ice cold PBS. Brains were removed, separated into right and left hemispheres and snap frozen, then stored at –80°C for later analysis.

### DP-460

Twenty SHRSP/JPD/UCAO rats were treated with DP-460 (Produced and tested by D-Pharma in Tel Aviv, Israel) beginning at 12 weeks of life, immediately after the UCAO surgery. Rats were placed on the JPD at the 12 week timepoint.

The DP-460 drug was dissolved in 5% dextrose and injected subcutaneously (every 3^rd^ day). Another group of 20 SHRSP/JPD/UCAO rats that were similarly prepared and treated with 5% dextrose served as a control group. An additional untreated group was SHRSP rats who were sham operated (n = 5). The SHRSP/JPD/UCAO animals were started on the JPD at the age of 12 weeks, following right unilateral carotid artery occlusion (UCAO) surgery.

Weight and blood pressure measurements were recorded throughout the study. Behavioral testing for learning/memory was performed during the 15^th^ week via the MWM. We used MRI modalities (T2, FA, ADC) to measure water content in the white matter and hippocampus prior to sacrifice at 16 weeks (4 weeks of drug treatment), as previously described.[15] These time points were selected based on a prior study of minocycline. Following sacrifice, animals were perfused with ice cold PBS and brain tissue collected. Experiments were done in duplicates, at two different intervals, with n = 10 rats/group to validate our findings.

### SB-3CT

Experiments with SB-3CT (Produced and tested by the Mobashery group in the University of Notre Dame, Notre Dame, Indiana) were carried out similarly to those with DP-460, beginning at 12 weeks of life, and immediately after the UCAO surgery. Rats were placed on the JPD at the 12 week timepoint.

The drug was administered intraperitoneally every other day at a dose of 10 mg/kg. The vehicle for SB-3CT consisted of 30% DMSO, 60% PEG-200 (Sigma-Aldrich, cat # 88440), and 10% sterile water. All animals in the study were sacrificed at the 20^th^ week timepoint due to preparation with DMSO, a reagent known to prolong lifespan by acting as a neuroprotectant (following 8 weeks of drug treatment).

### MMP-9 measurements

Vehicle (n = 10) and experimental (n = 10) rat brains were separated into left and right hemispheres and weighed after removing the olfactory bulb and the cerebellum/spinal cord. The samples were homogenized in modified (lacking EDTA) RIPA (Radio-Immunoprecipitation Assay) Lysis Buffer, in pre-calculated amounts based on weight. Each of the hemispheres was homogenized, then sonicated to lyse any remaining intact cells. The samples were centrifuged and the supernatant was extracted. The samples were run through the BCA protein assay (Thermo Scientific) to determine the total protein content. Levels of MMP-9 in the samples were assessed by gelation zymography, as described in detail elsewhere.[16] Briefly, proteins were separated electrophoretically on 10% polyacrylamide gels, washed twice in Zymogram Renaturation Buffer (Bio-Rad) and incubated for 96 hours at 37°C in Zymogram Development buffer (Bio-Rad). Gels were then rinsed in tap water and stained for 1 hour in Brilliant Blue R staining solution (Sigma Aldrich). Following the stain, the gels were rinsed twice in tap water and in 10% acetic acid and then incubated in fresh 10% acetic acid over two nights at room temperature. Gels were dried between Gel-Air Cellophane Support sheets (Bio-Rad) overnight. Dried gels were scanned on a transparency scanner (Epson V700) using the TWAIN capture feature of Photoshop (Adobe Inc.) and lytic bands were measured densitometrically with AlphaEase FC (Alpha Innotech).

### Statistical analysis

All data was confirmed by a Clinical Translational Science Center biostatistian at the University of New Mexico and all significance tests were performed at the 0.05 level of significance. Pairwise correlations between the measured variables (PT time, PT crosses, HIPP T2, CA1 T2, CC T2, EL time) were estimated and tested using Kendal’s Tau statistic, as all variable pairs exhibited roughly monotonic (but not necessarily linear) associations. MMP-9 measurement data was prepared using Prism (version 6 Graphpad Software Inc.) and expressed as Integrated Density Value of the lysed 92kDa bands on the gels. One-way ANOVA was performed to detect if any differences were present among the means of the 3 groups (treatment, vehicle, and control). T-tests were used for two group comparisons, as appropriate, for weight, blood pressure, MWM and MMP-9 level comparisons of drug and VEH groups.

## Results

**Fig 1:**
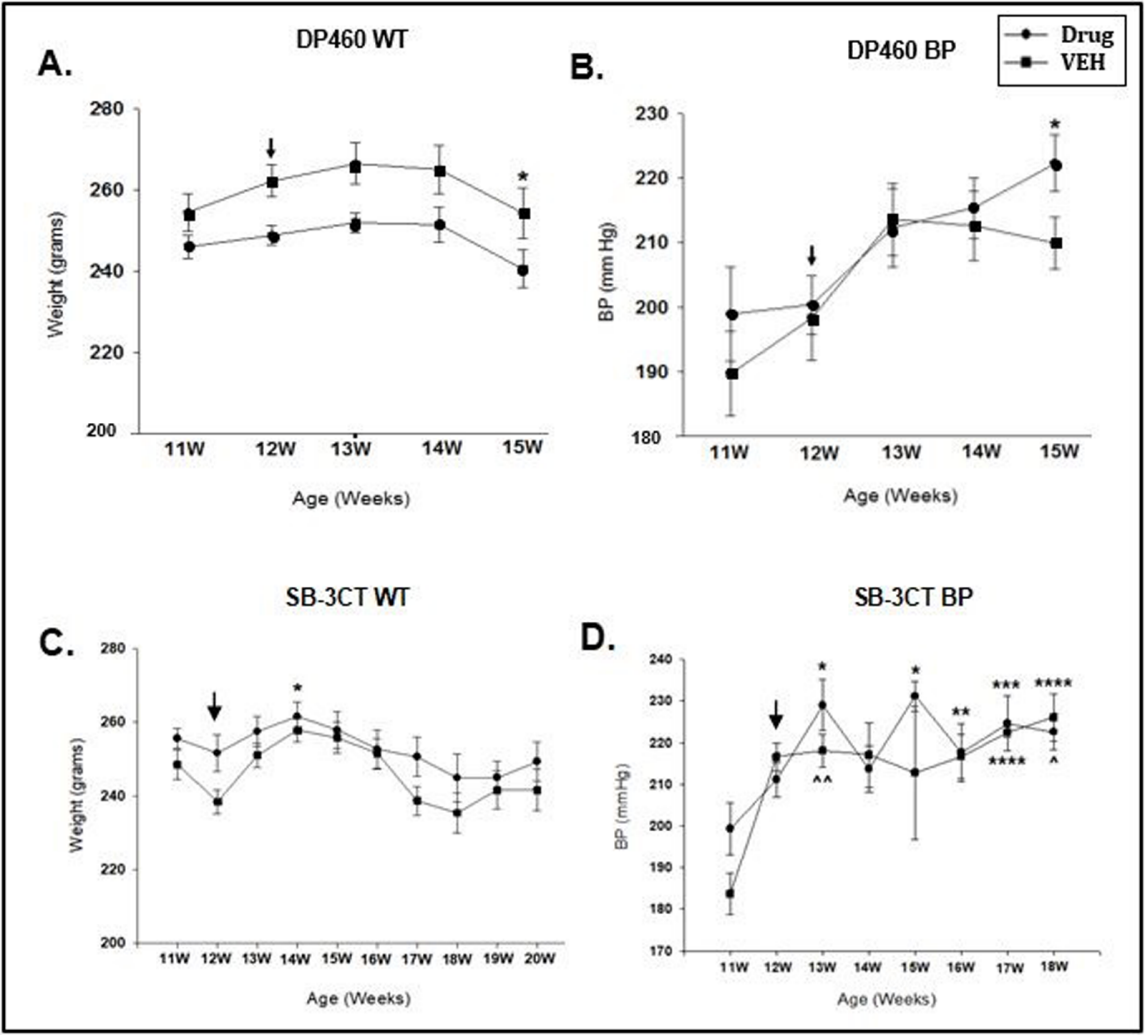
Distribution of WT and BP data for DP-460 and SB-3CT drug study animals. (A). Timecourse of WT distribution for SHRSP/JPD/UCAO treated with the DP-460 drug compared with 5% dextrose-treated control rats. Significantly lower WT was detected at 15 weeks in treated rats compared to the 13 week (*p = 0.004) and 14 week (*p = 0.011) VEH group. (B). Timecourse of BP distribution in SHRSP/JPD/UCAO treated with the DP-460 drug compared with 5% dextrose-treated control rats. BP was significantly elevated at 15 weeks in drug versus 11 weeks vehicle group (*p = 0.007). (C). SB-3CT WT distribution over 20 weeks of rat life compared to the VEH group. Higher WT was found at 14 weeks in the SHRSP/JPD/UCAO drug group compared to 18 weeks in the DMSO-treated VEH group (*p = 0.015). (D). A BP timecourse for SB-3CT comparison with DMSO-treated VEH shows several significant findings: drug at 13 week (*p<0.001), 15 week (*p<0.001), 16 week (**p = 0.050), 17 week (***p = 0.004) and 18 week (****p = 0.009) compared to 11 week VEH; VEH at 13 week (^^p = 0.043), 17 week (****p = 0.009), 18 week (^p = 0.002) compared to 11 week VEH. Data is based on drug (n = 20) and VEH (n = 5) groups for DP-460 and SB-3CT studies, respectively. The black arrow at the 12 week timepoint per graph represent the beginning of the JPD. Week (W).

The effect of the two drugs on blood pressure and weight in comparison to the appropriate controls is shown in **Fig 1**(refer to **S1 Fig** for a detailed table). DP-460 caused a decrease in weight at 15 weeks compared to 13 week and 14 week timepoints (p = 0.004, p = 0.011; **Fig 1A**) and an increase in blood pressure at 15 weeks (p = 0.007; **Fig 1B)** compared to the VEH group. SB-3CT increased weight at 14 weeks (p = 0.015; **Fig 1C**) and had a variable effect on blood pressure (drug at 13 week (p<0.001), 15 week (p<0.001), 16 week (p = 0.050), 17 week (p = 0.004) and 18 week (p = 0.009) compared to VEH; **Fig 1D**).

MRI measurements of T2, diffusion as measured with fractional anisotropy (FA), apparent diffusion coefficient (ADC) in the gray (hippocampus) and white matter (corpus callosum, external capsule) regions are shown in **Table 1**. Neither DP-460 nor SB-3CT had a significant effect on any of the MRI variables.

**Table 1:** MRI modalities are unchanged between treatment and control groups for DP-460 and SB-3CT studies.

	Corpus Callosum			External Capsule			Hippocampus		
	T2-MRI	FA	ADC	T2-MRI	FA	ADC	T2-MRI	FA	ADC
DP-460	2.80±0.12	0.26±0.01	0.00083± 0.0000078	2.53±0.12	0.25±0.02	0.00085±0.000019	2.75±0.17	0.39±0.01	0.0009± 0.000012
DP-460 VEH	2.90±0.15	0.27±0.01	0.00082± 0.000007	2.72±0.13	0.25±0.02	0.00083±0.000014	2.50±0.19	0.41±0.01	0.00089±0.000018
DP-460 CONT	2.38±0.06	0.26±0.01	0.00082± 0.000014	2.38±0.03	0.24±0.03	0.00081±0.000013	2.45±0.13	0.40±0.02	0.00089±0.000015
SB-3CT	2.75±0.19	0.31±0.03		2.70±0.21			2.73±0.30		
SB-3CT VEH	2.85±0.21	0.25±0.02		2.89±0.23			2.62±0.16		
SB-3CT CONT	3.84±0.96	0.31±0.03		3.08±0.39			2.90±0.23		

Structural brain imaging of normalized T2-MRI intensity ratios and diffusion tensor imaging parameters (FA, ADC) are not significantly different between DP-460-treated, VEH-treated and untreated, naïve control groups. Data analyses of T2-MRI and FA imaging parameters failed to show significant differences between SB-3CT-treated, VEH-treated and naïve control groups. Data is represented as mean±SE and analyzed using a one-Way ANOVA. None of the p-values associated with the one-way ANOVA tests were found to be significant at the 0.05 level.

**Fig 2:**
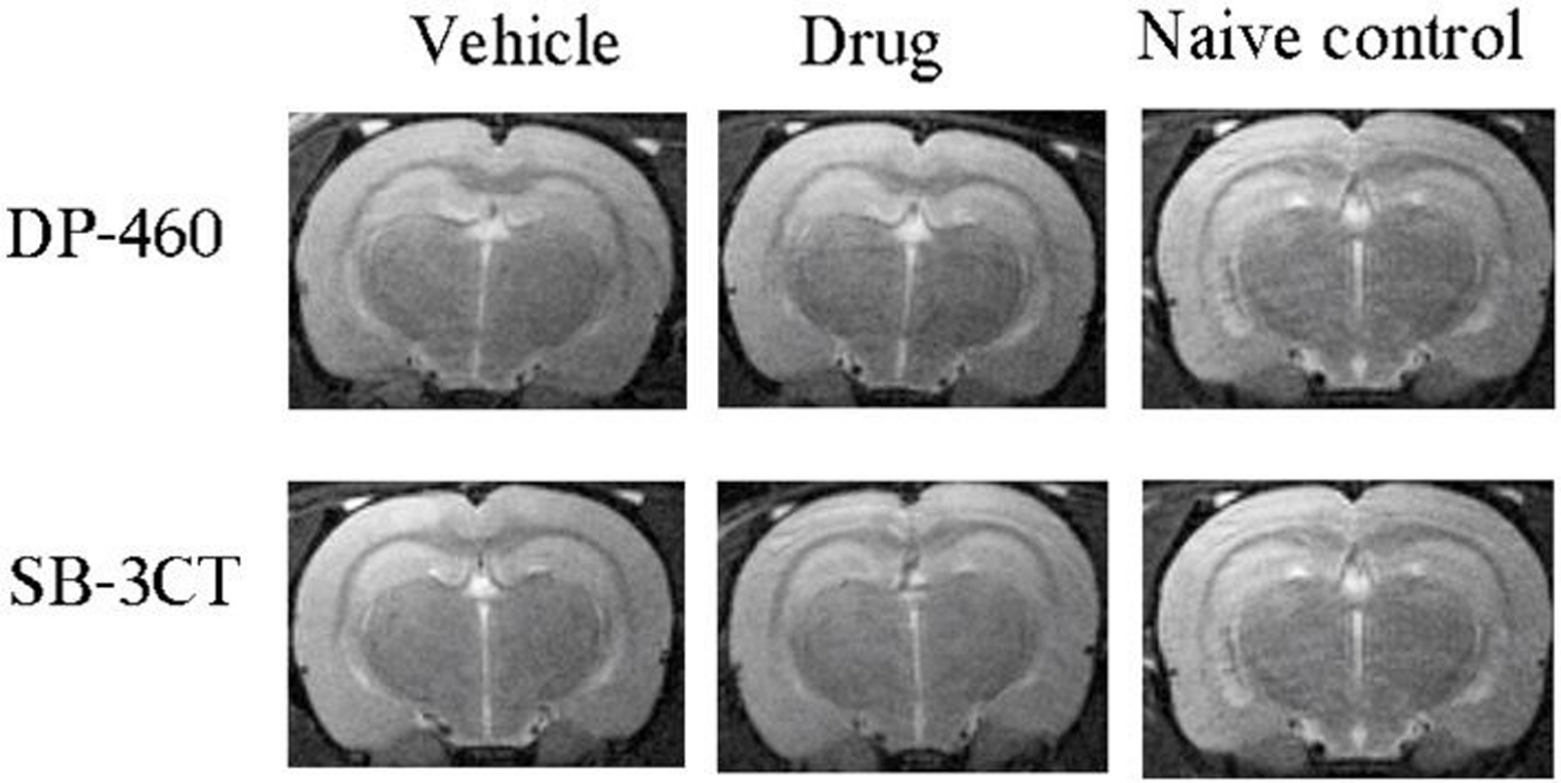
Brains are unchanged with DP-460 and SB-3CT treatments. Representative T2-MRI images were obtained at 16 weeks of life for DP-460 and 20 weeks of life for SB-3CT. No differences were found among treatment and control groups (n = 10/group).

Representative T2-MRI images of DP-460 and SB-3CT brains are shown in **Fig 2**. No visible changes were observed among brains from the DP-460, SB-3CT and naïve control groups.

**Fig 3:**
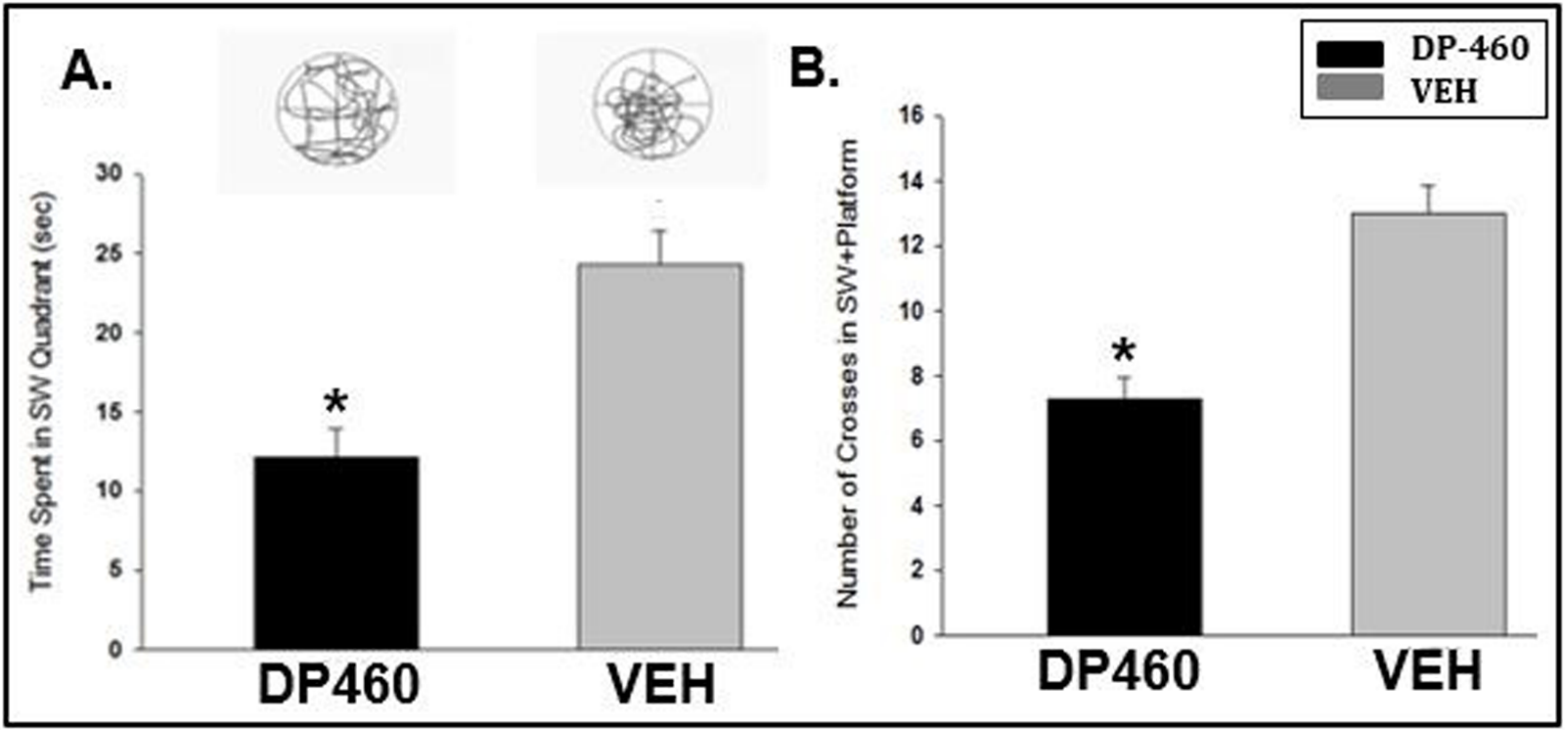
DP-460 impairs learning and memory in the Morris water maze. A significant decrease in time spent in seconds for the DP-460 group (A) and the number of crosses (B) in the Southwest (SW) quadrant during the probe trial (*p<0.001), indicates poorer performance for the drug-treated animals. Corresponding trace diagrams reveal the swimming path of the rat to the goal quadrant.

Unexpectedly, behavioral studies in the MWM studies showed an impaired ability to learn with DP-460 and no effect on learning with SB-3CT. The time spent (**Fig 3A**) and number of crosses (**Fig 3B**) in the Southwest quadrant was significantly less with DP-460 treatment, indicating that the drug impaired performance. Treatment with SB-3CT had no effect on learning, likely due to its distinct mechanism of action compared to DP-460. Refer to **S2 Fig** for a detailed table.

For the DP-460 study, a modest positive association was found between the time spent in the probe trial of the MWM and hippocampus CA1 hyperintensities (Kendall’s Tau: 0.31, p = 0.02), while a modest negative association was found between the number of crosses of the goal platform in the probe trial and corpus callosum hyperintensities (Kendall’s Tau: -0.37, p<0.01). For the SB-3CT study, a moderate positive association was found between total hippocampus hyperintensities and escape latency, the time it takes to find the goal platform in the MWM (Kendall’s Tau: 0.47, p = 0.03; **Table 2**).

**Table 2:** Significant Kendall’s Tau correlations for DP-460 and SB-3CT drug studies.

Drug Study	Variable 1	Variable 2	Kendall’s Tau	p-Value
**DP-460**	PT Time	CA1 T2	0.31	0.02
**DP-460**	PT Crosses	CC T2	-0.37	<0.01
**SB-3CT**	HIPP T2	EL Time	0.47	0.03

Calculations of Kendall’s Tau detected several significant correlations between two measured variables in the drug studies. CA1-cornu Ammon (a hippocampal subregion); CC-corpus callosum; EL-escape latency; HIPP-hippocampus; MWM-Morris Water Maze; PT-probe trial; T2-T2-weighted imaging (transverse relaxation time in MRI).

**Fig 4:**
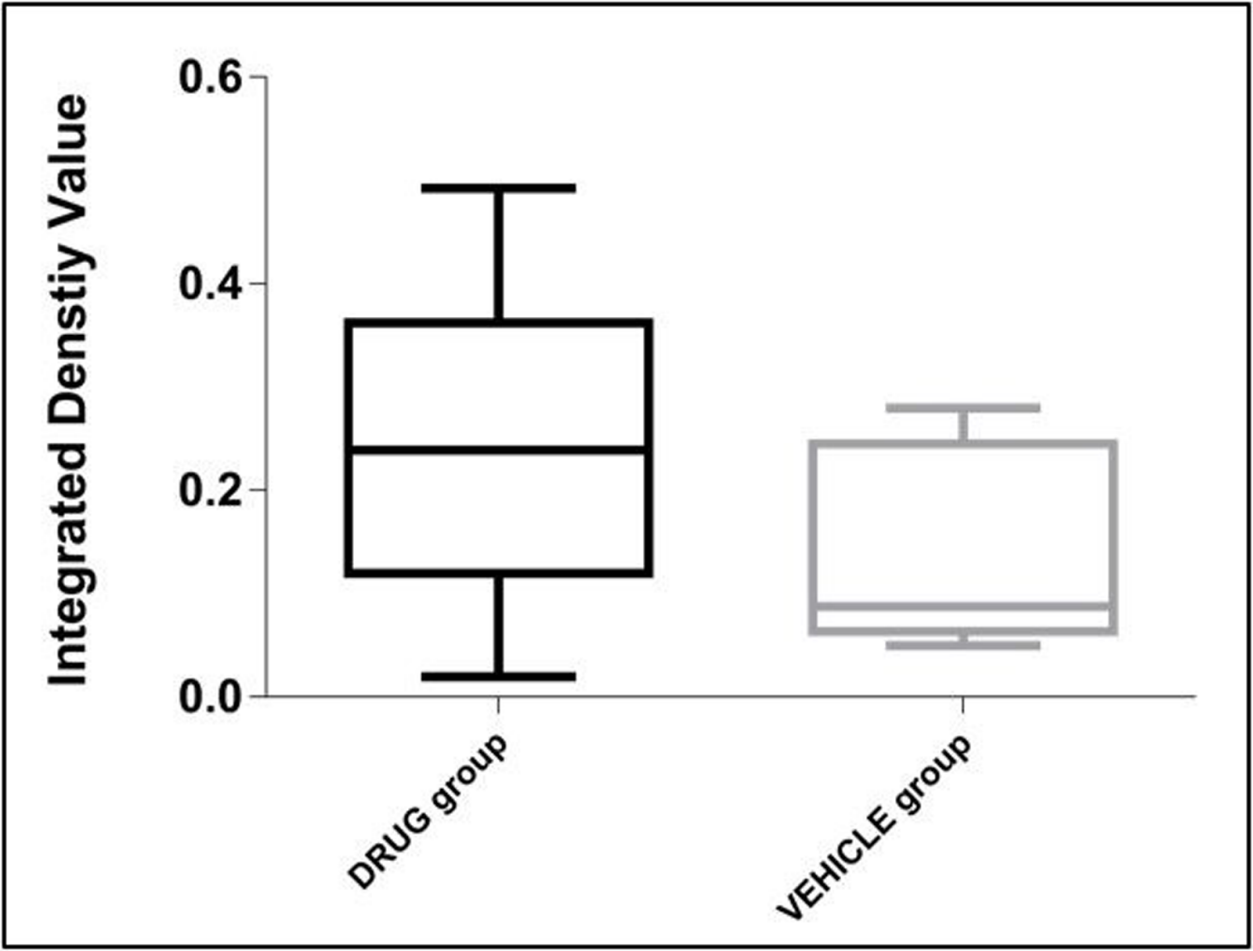
Gel zymography for MMP-9 levels. No significant differences were observed for MMP-9 levels between DP-460 drug and VEH groups. MMP-9 levels were expressed as integrated density value of 92kDa lytic bands on zymographic gels. Data is represented as box plots, with the median and interquartile ranges (n = 10 rats per group).

We used gel zymography to determine the level of MMP-9 in the tissues of the animals treated with DP-460, which showed the negative impact on learning. The levels of MMP-9 detected in the post-treatment tissues were unchanged by the drug (p = 0.067, **Fig 4**; refer to **S3 Fig** for a detailed table). Since SB-3CT failed to affect either the MRI variables or the behavior, measurements of MMPs in the tissue were not obtained.

## Discussion

A number of studies have shown that MMPs have a detrimental effect in neurological diseases and that MMP inhibitors can help reduce the injury.[10, 17, 18] During an acute stroke, there is disruption of the BBB with breakdown of extracellular matrix and tight junction proteins by MMPs, particularly MMP-9.[8] In an animal model of chronic stroke with features similar to patients with VCID, treatment with two relatively selective MMP-9 inhibitors failed to show improvement in MRI parameters and behavioral measures. DP-460 had a detrimental effect on learning, while SB-3CT had no effect. This unexpected result suggests that minocycline’s beneficial action in our previous studies was not related to inhibition of MMP-9, but rather to a broader, pleiotropic effect on inflammation that could involve suppression of microglia and multiple MMPs.[19–22] The lack of benefit by both MMP-9 inhibitors, DP-460 and SB-3CT, which cross the blood-brain barrier, suggests that MMP-9 is not the major factor driving the white matter damage in the SHRSP model of vascular cognitive impairment.[23]

There are several explanations for the lack of an effect of either agent on the white matter damage measured with MRI and behavior measured by the MWM. It is possible that MMP-9 is not the major MMP involved in chronic ischemia as opposed to acute cerebral ischemia, where it is a major factor. Other MMPs are expressed in the brain cells of the SHRSP model. Stromelysin-1 (MMP-3) is produced by microglia, pericytes, and macrophages. Astrocytes produce MMP-2. Neurons have MMP-10.[24, 25] SB-3CT is a slow-binding inhibitor for MMP-2 and MMP-8 and linear-competitive inhibitor of MMP-14. It is very potent for MMP-2 and MMP-9.[14] The profile for DP-460 MMP inhibition may be broader than the SB-3CT compound. Thus, other MMPs may be more important contributors to brain damage than MMP-9 alone.

The two agents have different mechanisms of action: DP-460 chelates the active-site zinc ion of the MMP within the catalytic domain,[26] while SB-3CT binds the active site and uses the catalytic machinery of the enzyme to undergo a reaction that generates a species, resulting in tight-binding inhibition. In light of the fact that this reaction has been documented only within the active-site of gelatinase (MMP-2 and MMP-9), the compound affords high selectivity for targeting of these enzymes.[14] The impairment of learning induced by DP-460 suggests that it had a central action, albeit a detrimental one, possibly due to its lack of high selectivity for MMP-9.

While this study was underway, several reports appeared that could shed light on the negative effects of MMP-9 inhibition in chronic stroke. Other investigators found that MMP-9 inhibitors blocked the formation of perineuronal nets involved in formation of synapses.[27, 28] These synaptic nets are critical for consolidation of learning. Thus, a probable explanation of the negative effect of DP-460 on learning in the MWM is that MMP-9 is necessary for memory consolidation and the DP-460 drug lacked high selectivity for this MMP isoform. MMP-9 is necessary for synaptic remodeling, most likely through its action on perineuronal nets.[27–29] In those studies, the investigators found impaired learning in the MWM in rats treated with an MMP-9 inhibitor, DP-b99, which provides the most plausible explanation of the impaired learning seen in our study.[30]

In studies of humans with VCID, we and others have shown elevation of several MMPs in CSF.[31, 32] MMP-2 is reduced and MMP-9 elevated in the CSF of patients with VCID. MMP-10 is increased in CSF of patients with Alzheimer’s disease and MMP-3 is elevated in mixed dementia (unpublished data).

There is increasing evidence that MMP-9 is important in the formation of synapses after an injury.[33] This has been demonstrated by several groups of investigators, who have shown that blocking MMP-9 reduces synaptic plasticity and hinders learning and memory. Neuronal MMP-9 has been implicated in synaptic plasticity through the regulation of dendritic spine structure and excitatory synapse function.[34, 35] Additional evidence suggests that inhibition of MMP-9 could block hippocampal neurogenesis.[36]

Our results show that drugs that affect acute ischemic injury may not be suitable for chronic ischemia, and that while MMPs have a detrimental effect in the early stages of an injury, they are important in the later recovery phases. Furthermore, these results suggest that the anti-inflammatory effects of minocycline, which was beneficial in reducing white matter injury and cognitive deficits in the same animal model, must be acting through other mechanisms than direct inhibition of MMP-9. More research will be needed to characterize the sequence of MMP expression over time and the production of other factors that could contribute to the white matter injury. A general finding with the MMP inhibitors is that they have both beneficial and detrimental actions, depending on the timing of their use after the injury.[37, 38] Since minocycline showed greater efficacy, the role of inflammation appears to be critical, and the design and testing of agents that act on the MMPs at the earlier inflammatory stage, without interfering with the recovery process, are now needed.

## Supporting information

S1 Fig Distribution of WT and BP data for DP-460 and SB-3CT drug study animals. Timecourse of data distribution for WT and BP for the DP-460 and SB-3CT drugs are presented as mean±SE.
(TIFF)

S2 Fig DP-460 impairs learning and memory in the Morris water maze. Data distribution for the probe trial duration of time spent in the SW quadrant (n = 20 rats/group) and the number of crosses in the SW quadrant (n = 10 rats/group) are presented for the DP-460 drug study.
(TIFF)

S3 Fig Gel zymography for MMP-9 levels. Data distribution for DP-460 drug study MMP-9 levels as compared to the VEH group. Note that some data values were below the detection threshold of assay, and therefore omitted.
(TIFF)
