# Supplementary figures and images for: MMP-9 inhibitors impair learning in spontaneously hypertensive rats
Source: PLoS One. 2018 Dec 11;13(12):e0208357. doi: 10.1371/journal.pone.0208357 (PMC6289411; doi:10.1371/journal.pone.0208357)

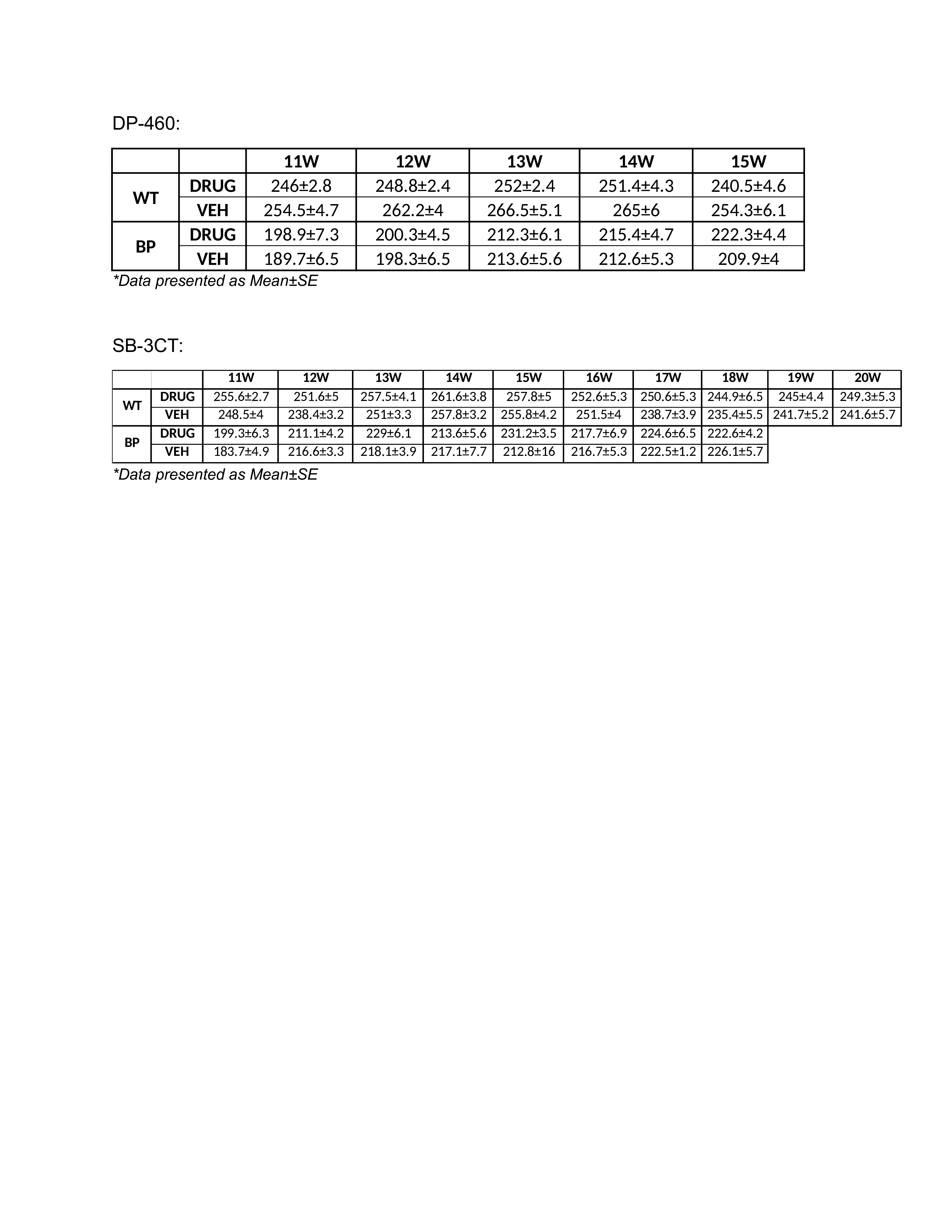

Supplement: S1 Fig — Timecourse of data distribution for WT and BP for the DP-460 and SB-3CT drugs are presented as mean±SE. (TIFF) [file pone.0208357.s001.tiff]

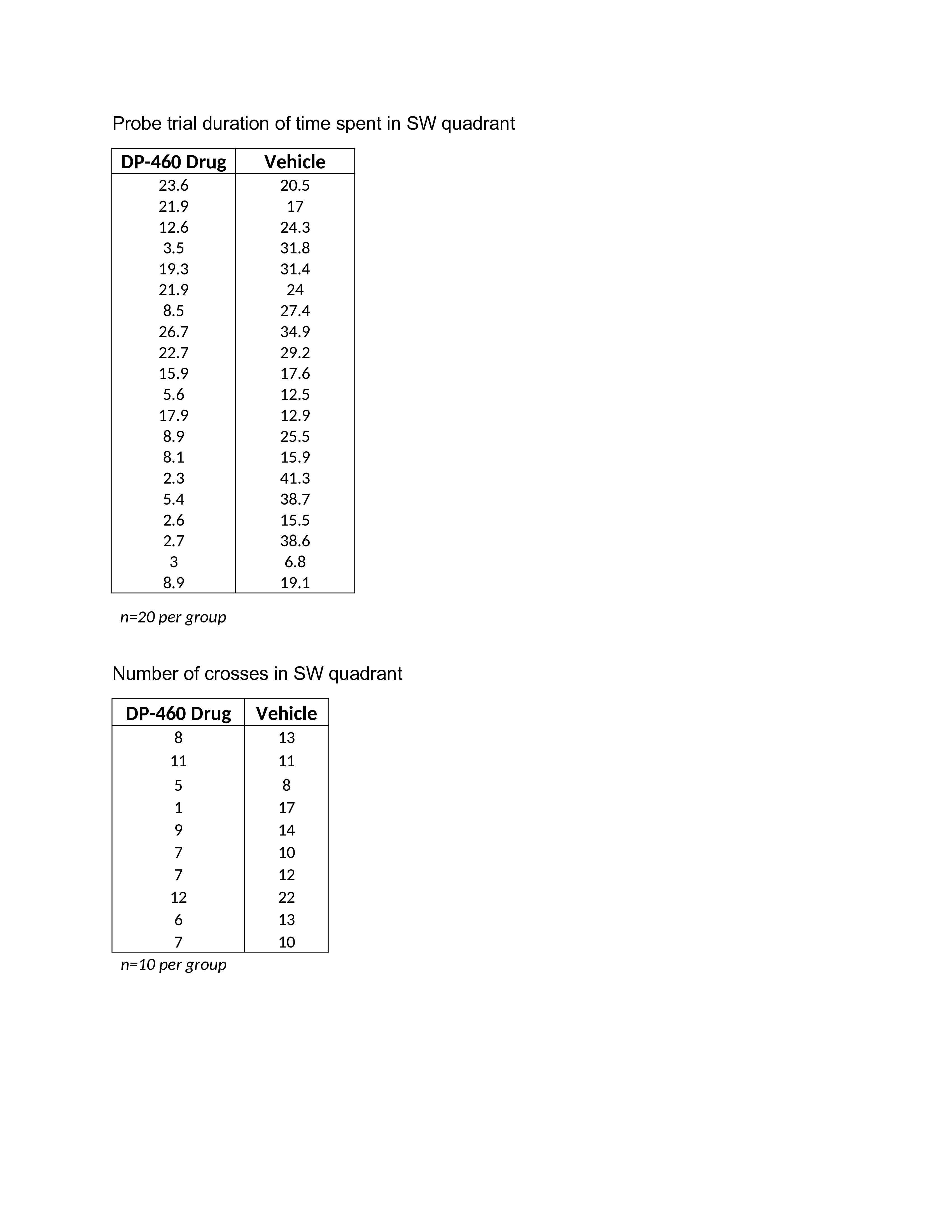

Supplement: S2 Fig — Data distribution for the probe trial duration of time spent in the SW quadrant (n = 20 rats/group) and the number of crosses in the SW quadrant (n = 10 rats/group) are presented for the DP-460 drug study. (TIFF) [file pone.0208357.s002.tiff]

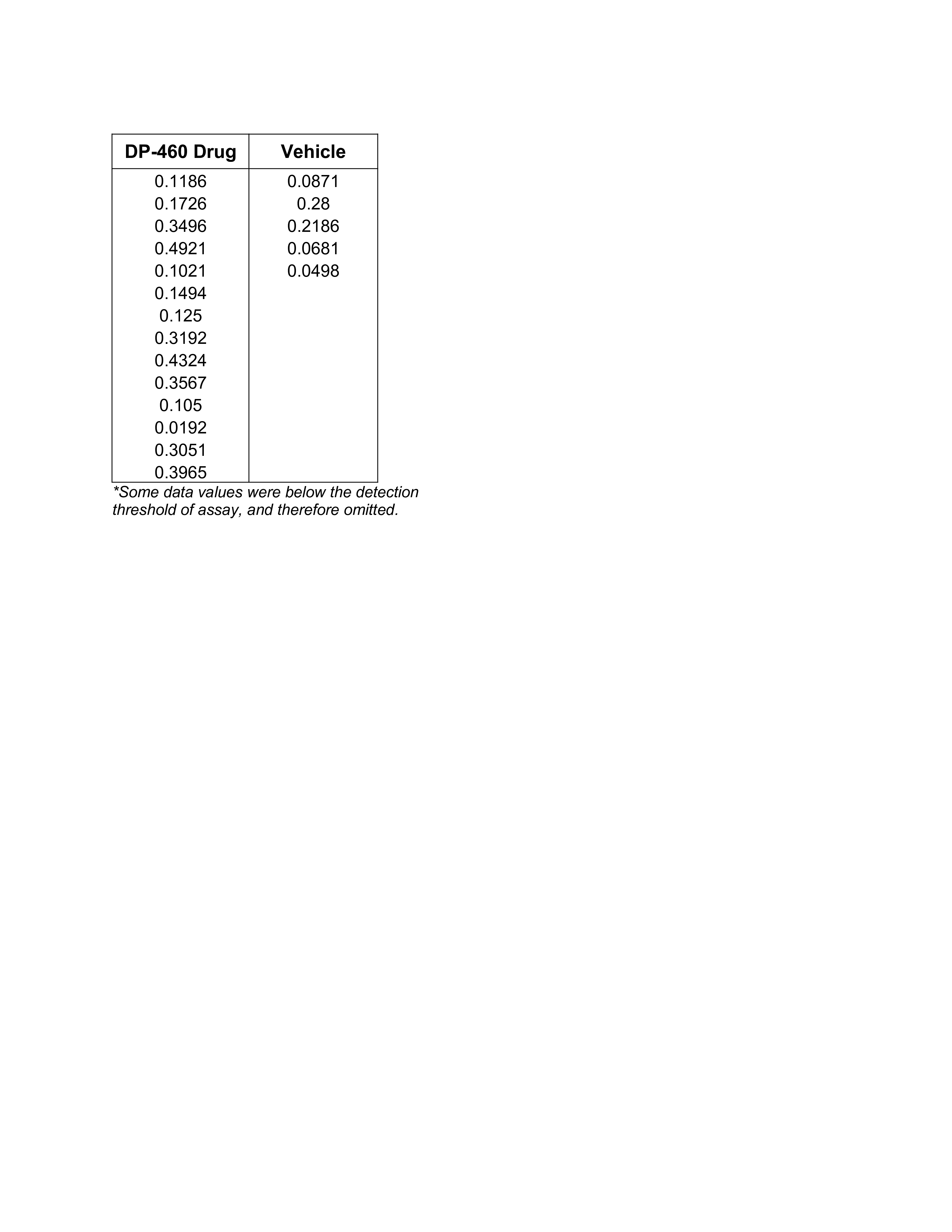

Supplement: S3 Fig — Data distribution for DP-460 drug study MMP-9 levels as compared to the VEH group. Note that some data values were below the detection threshold of assay, and therefore omitted. (TIFF) [file pone.0208357.s003.tiff]
